# Changing Clothing

**Published:** 1879-10

**Authors:** 


### CHANGING CLOTHING.

Winter is the harvest of death to those who have reached three-score, because thicker clothing is not put on soon enough. The rule should be to wear woolen flannel next the skin, limbs and body ; after September 1st increasing in thickness to Dec. 1st. Then the old, the very young, the women, and all in feeble health should avoid going out of doors until after breakfast, and should keep in the house after sun-down, unless when the weather is still and dry, and crisp.

India rubber shoes should be worn by all such from November first when going out for a walk, or visit or shopping : dampness is effectually excluded, and the heat is confined to the feet and the cold excluded very much better than by leather. On coming into the house, take off the shoes, hold the stocking feet to the fire, and put on a pair of warmed slippers : this is comfort ; its neglect is followed by bad colds and spells of sickness.

